# Identification of Veterans With PTSD Based on EEG Features Collected During Sleep

**DOI:** 10.3389/fpsyt.2020.532623

**Published:** 2020-10-30

**Authors:** Srinivas Laxminarayan, Chao Wang, Tatsuya Oyama, J. David Cashmere, Anne Germain, Jaques Reifman

**Affiliations:** ^1^Department of Defense Biotechnology High Performance Computing Software Applications Institute, Telemedicine and Advanced Technology Research Center, United States Army Medical Research and Development Command, Fort Detrick, MD, United States; ^2^The Henry M. Jackson Foundation for the Advancement of Military Medicine, Inc., Bethesda, MD, United States; ^3^Department of Psychiatry, University of Pittsburgh School of Medicine, Pittsburgh, PA, United States

**Keywords:** electroencephalography, sleep-stage independent, classification, sleep, PTSD, spectral power, synchrony

## Abstract

**Background:** Previously, we identified sleep-electroencephalography (EEG) spectral power and synchrony features that differed significantly at a population-average level between subjects with and without posttraumatic stress disorder (PTSD). Here, we aimed to examine the extent to which a combination of such features could objectively identify individual subjects with PTSD.

**Methods:** We analyzed EEG data recorded from 78 combat-exposed Veteran men with (*n* = 31) and without (*n* = 47) PTSD during two consecutive nights of sleep. To obviate the need for manual assessment of sleep staging and facilitate extraction of features from the EEG data, for each subject, we computed 780 stage-independent, whole-night features from the 10 most commonly used EEG channels. We performed feature selection and trained a logistic regression model using a *training* set consisting of the first 47 consecutive subjects (18 with PTSD) of the study. Then, we evaluated the model on a *testing* set consisting of the remaining 31 subjects (13 with PTSD).

**Results:** Feature selection yielded three uncorrelated features that were consistent across the two consecutive nights and discriminative of PTSD. One feature was from the spectral power in the delta band (2–4 Hz) and the other two were from phase synchronies in the alpha (10–12 Hz) and gamma (32–40 Hz) bands. When we combined these features into a logistic regression model to predict the subjects in the *testing* set, the trained model yielded areas under the receiver operating characteristic curve of at least 0.80. Importantly, the model yielded a *testing*-set sensitivity of 0.85 and a positive predictive value (PPV) of 0.31.

**Conclusions:** We identified robust stage-independent, whole-night features from EEG signals and combined them into a logistic regression model to discriminate subjects with and without PTSD. On the *testing* set, the model yielded a high sensitivity and a PPV that was twice the prevalence rate of PTSD in the U.S. Veteran population. We conclude that, using EEG signals collected during sleep, such a model can potentially serve as a means to objectively identify U.S. Veteran men with PTSD.

## Introduction

Sleep disturbances are a hallmark of posttraumatic stress disorder (PTSD) (1). For this reason, previous studies have analyzed electroencephalography (EEG) data from overnight sleep-polysomnography (PSG) recordings to identify differences in sleep patterns between groups of subjects with and without PTSD (2–5). Motivated similarly, but with the intent to find differences that are reproducible, we recently identified EEG spectral powers that discriminate combat-exposed Veterans with and without PTSD at the group-average level (6). Specifically, we split the sleep-study data into a set for initial discovery and a set for testing reproducibility of our findings. In that study, we found that the features that showed group-level differences were consistent across two consecutive nights in the initial discovery set and, importantly, that these findings were largely reproducible on the held-out test set. More recently, analyzing the data from the same study using a similar procedure, we found that the synchrony of EEG signals between channel pairs [the average phase difference between two time-series signals over a given time interval (7)] could also significantly discriminate the two groups (8). Another recent study by Modarres et al. (9)—the only publication to date that investigated synchrony between EEG channels in PTSD subjects during sleep—also reported group-level differences, although they did not assess the reproducibility of their results across multiple nights or in an independent dataset. Together, these findings suggest that EEG spectral power and synchrony features can distinguish differences between groups of subjects with and without PTSD (2–5).

The natural next step is to investigate whether these and similar features can be used to diagnose PTSD at the individual level. Current methods of PTSD diagnosis are subjective, relying on a clinician's judgement and a patient's self-report in questionnaires, such as the clinician-administered PTSD scale (CAPS) (10) and the insomnia severity index (ISI) (11). In contrast, an objective means to identify subjects with PTSD would aid clinicians in adjudicating true cases with increased specificity and enable them to track the responses of patients to treatment, while providing the opportunity to shed light on the neurophysiological mechanisms of PTSD (12).

Here, encouraged by the promising findings in the above-mentioned group-difference studies (6, 8), we aimed to assess whether a multivariate classifier, developed using EEG spectral power and synchrony features, could objectively identify individual subjects with PTSD. To this end, we analyzed EEG data recorded from 78 combat-exposed Veteran men with (*n* = 31) and without (*n* = 47) PTSD during two consecutive nights of sleep. Following our recent work, we developed a multivariate classifier using a *training* set, which consisted of the first 47 consecutive subjects (18 with PTSD) of the study, and evaluated the classifier on the *test* set, which consisted of the remaining 31 subjects (13 with PTSD), in order to assess the reproducibility of our findings. In this procedure, we used stage-independent, whole-night features computed on data from the 10 most commonly used EEG channels to facilitate comparison of results across laboratories, because most PSG studies record EEG data from 10 or fewer channels.

## Methods

We recruited combat-exposed Veterans who provided written informed consent in accordance with the protocol approved by the University of Pittsburgh Institutional Review Board (Pittsburgh, PA) and the U.S. Army Medical Research and Development Command Human Research Protection Office (Ft. Detrick, MD). We excluded subjects with any of the following conditions from the study: a current diagnosis of untreated severe depression, psychotic or bipolar disorder, substance or alcohol abuse in the previous 3 months, or sleep disorders other than insomnia or nightmares. It should be noted that we did not exclude subjects with a prior history of alcohol consumption, because doing so would have greatly reduced the sample size and, importantly, the generalizability of our results to Service member populations, in which alcohol consumption is common. All subjects were free of any sleep-related medication for at least 2 weeks prior to enrollment in the study. Before their arrival at the laboratory, we assessed subjects' habitual sleep patterns for 10 consecutive days using a sleep diary (Table 1). During this time, we also instructed them to take no more than two cups of coffee per day (or the equivalent caffeine dose) and limit their alcohol intake to two drinks per day over a 2-week period before the study. We also assessed the presence and severity of PTSD via the CAPS (10), the presence of alcohol use disorder in the past month, sleep quality via the Pittsburgh sleep quality index (13) and the ISI (11), and self-reported measures of depression via a patient health questionnaire (14).

**Table 1:** Clinical characteristics and sleep-diary variables for the 78 combat-exposed Veteran men in our study.

**Variable**	**PTSD (n = 31) Mean (SD)**	**Non-PTSD (n = 47) Mean (SD)**	**Group comparison *p*-value^a^**
Age (y)	31.3 (4.7)	32.8 (6.2)	0.358
Sleep diary^b^			
Time in bed (min)	453.0 (100.6)	465.0 (55.3)	0.580
Total sleep time (min)	414.3 (77.0)	444.1 (52.5)	**0.035**
Sleep efficiency (%)	92.8 (9.5)	95.6 (3.4)	**0.004**
Sleep latency (min)	27.8 (17.1)	10.0 (5.9)	**<0.001**
CAPS	51.4 (16.8)	8.6 (7.9)	**<0.001**
Hyperarousal	19.0 (7.1)	3.3 (4.0)	**<0.001**
Intrusion	10.7 (5.8)	0.6 (1.8)	**<0.001**
Avoidance	16.9 (8.8)	1.7 (3.5)	**<0.001**
Current^c^ AUD (n)	2	0	–
Past^d^ AUD (n)	17	10	–
PSQI	8.9 (2.8)	4.1 (2.4)	**<0.001**
ISI	14.2 (4.8)	3.8 (4.2)	**<0.001**
PHQ-9	5.8 (2.6)	1.4 (2.5)	**<0.001**

a *Wilcoxon rank-sum test, bold values indicate p < 0.05*;

b *PTSD, n = 30*;

c *Present in the past month*;

d *Absent in the past month*.

*AUD, alcohol use disorder; CAPS, Clinician-Administered PTSD Scale; ISI, Insomnia Severity Index; PHQ-9, Patient Health Questionnaire-9; PSQI, Pittsburgh Sleep Quality Index; SD, standard deviation*.

Subjects spent two consecutive nights and days in the University of Pittsburgh Medical Center's sleep laboratory. On Night 1, they arrived at 20:00 and were fitted with a PSG system, which consisted of a 64-channel high density-electroencephalography (hd-EEG) montage [HydroCel Geodesic Sensor Net (without sponge inserts); Electrical Geodesics Inc., Eugene, OR] and bipolar channels for submentalis electromyogram signals. Subjects were allowed to sleep undisturbed from 23:00 until 07:00, while we recorded their EEG data. On the morning of the next day, we removed the PSG system and asked the subjects to perform multiple tests to assess daytime alertness and cognitive functions. At 21:00, we refitted the subjects with the PSG system and repeated the same procedures on Night 2 and the following day until their discharge at 20:00.

Among the 85 subjects who completed the study, 37 (six women) met the diagnostic criteria for PTSD and 48 did not (one woman). We excluded all seven women from our analysis to avoid confounding effects due to sex differences (15). The remaining 78 men (31 with PTSD), who ranged from 24 to 51 years of age, comprised our study population (Table 1). We split this sample into a *training* set comprising the first 47 consecutive subjects of the study (18 with PTSD) for model development and a *test* set comprising the remaining 31 subjects (13 with PTSD) for assessing model performance.

### EEG Preprocessing and Feature Computation

We recorded hd-EEG data referenced to the linked mastoids at a sampling rate of 250 Hz. We visually scored sleep stages in 30-s epochs according to the criteria of the American Academy of Sleep Medicine (16). Table 2 shows the sleep architecture parameters for the study population.

**Table 2:** Sleep architecture measures for subjects with and without PTSD during two consecutive nights of sleep at the University of Pittsburgh sleep laboratory.

**Measure**	**Night 1**		**Night 2**	
	**PTSD (*n* = 31)**	**Non-PTSD (*n* = 47)**	**PTSD (*n* = 31)**	**Non-PTSD (*n* = 47)**
Total sleep time (min)	406.4 (36.1)	411.4 (34.3)	416.5 (27.1)	428.6 (35.3)
Sleep efficiency (%)	84.6 (27.8)	85.7 (7.9)	86.7 (5.6)	89.5 (7.4)
Stage N1 (%)	11.4 (4.8)	10.9 (5.6)	9.7 (3.8)	8.6 (4.6)
Stage N2 (%)	57.6 (7.4)	55.7 (7.2)	55.3 (7.2)	53.3 (6.5)
Stage N3 (%)	8.8 (6.8)	12.6 (7.4)	10.9 (5.9)	13.8 (7.2)
REM (%)	22.3 (5.7)	20.8 (5.3)	24.2 (5.6)	24.3 (5.9)

*Values within parentheses denote standard deviations*.

*REM, rapid eye movement sleep; N1, N2, and N3, non-REM stages of sleep*.

*None of the Wilcoxon rank-sum tests were significant at the p < 0.05 level, when we compared each of the measures between PTSD and non-PTSD, for each night*.

We applied a band-pass filter to preserve the EEG signals within the bandwidth of interest (0.5–50.0 Hz), while suppressing noise in frequency bands outside this range. Next, to minimize the impact of muscle movement in the EEG data, we segmented the data in each EEG channel into 5-s epochs and rejected transient, high-frequency activity whenever the power in the 26.0–50.0 Hz band of each epoch exceeded its moving median value over a 3-min window by a factor of four, as previously described (17, 18). Further, to eliminate artifacts due to body and head movement as well as poor electrode contact in each EEG channel, we removed the 5-s epochs for which the power in the 4.0–50.0 Hz band exceeded the whole-night median by a factor of six (6).

We computed three types of frequency domain EEG features—two to capture the mean and the coefficient of variation (the ratio between the standard deviation and the mean) of the log EEG power spectrum for each channel, and a third to capture the phase synchrony between pairs of EEG channels (with 1 denoting perfect synchrony and 0 denoting no synchrony) (7). We computed each of these features over the following 12 frequency bands spanning 0.5 to 40 Hz: 0.5–1 Hz (slow oscillations); 1–2 Hz [low delta (Lδ)]; 2–4 Hz [high delta (Hδ)]; 4–6 Hz [low theta (Lθ)]; 6–8 Hz [high theta (Hθ)]; 8–10 Hz [low alpha (Lα)]; 10–12 Hz [high alpha (Hα)]; 12–14 Hz [low sigma (Lσ)]; 14–16 Hz [high sigma (Hσ)]; 16–24 Hz [low beta (Lβ)]; 24–32 Hz [high beta (Hβ)]; and 32–40 Hz [low gamma (Lγ)].

### Feature Computation

**Figure 1:**
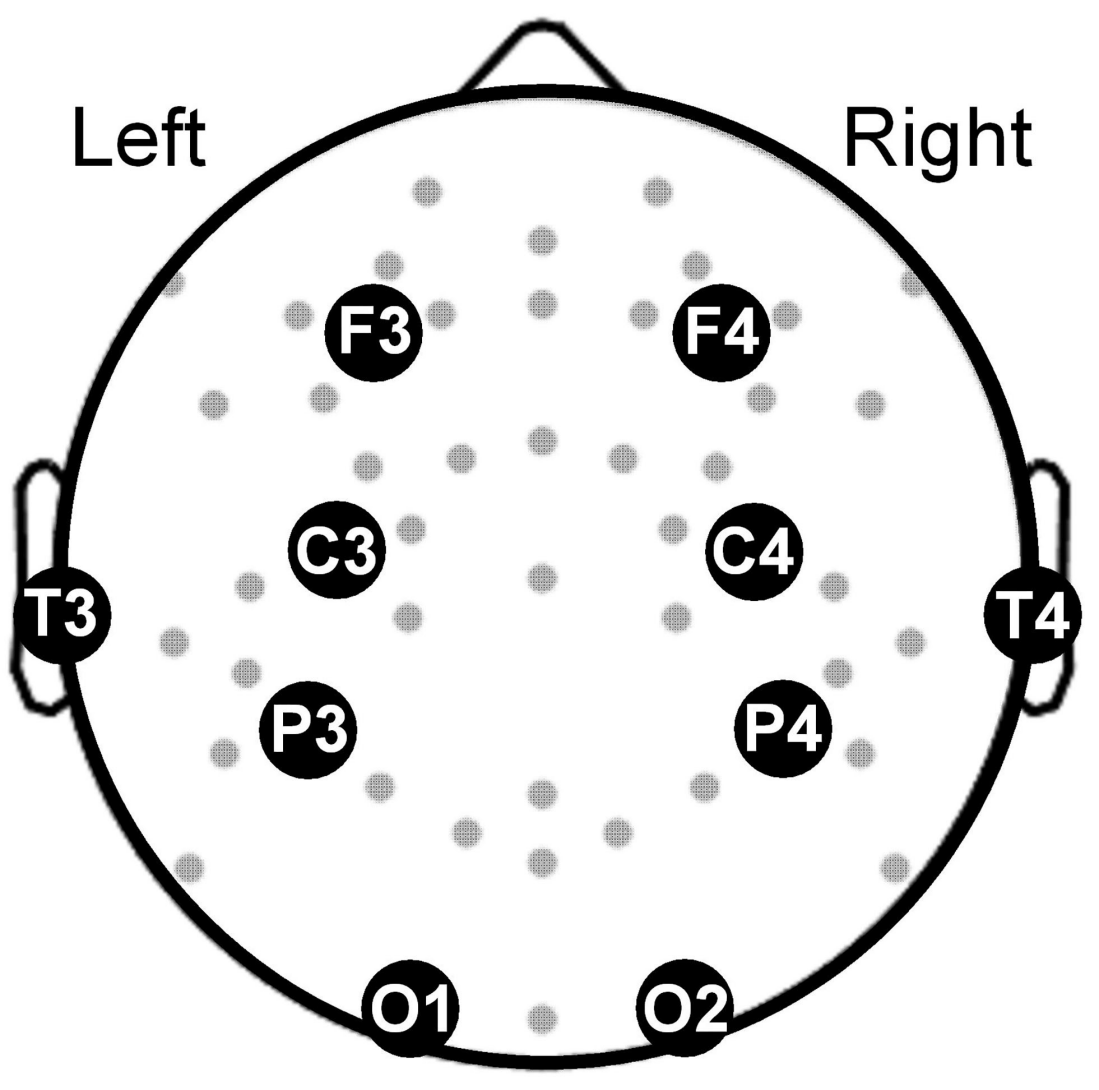
Topographical map showing the electroencephalography channels (gray dots) covering the brain. Large filled circles along with abbreviated names mark the locations of the 10 channels used in our analysis. F3 and F4: frontal channels; C3 and C4: central channels; T3 and T4: temporal channels, P3 and P4: parietal channels; O1 and O2: occipital channels.

Our objective was to develop a multivariate classifier that discriminates subjects with and without PTSD. As such, we aimed to reduce false associations due to temporal variations in the aforementioned features over the 8-h sleep period by studying their values averaged across the entire night disregarding sleep-stage specific information. Consequently, the averaged feature values contained the most information from the longest sleep stage, namely, the non-rapid eye movement (NREM) stage, which constituted more than 53% of the 8-h sleep period in both nights (Table 2). Furthermore, to increase the generalizability of our results to other sleep studies, we restricted our analyses to the 10 most commonly used EEG channels that cover the whole brain (Figure 1). Thus, for each subject, we computed a total of 780 whole-night features independent of sleep stage: 120 log powers (LP; 10 channels × 12 frequency bands), 120 coefficients of variation (LCV), and 540 phase synchronies [the weighted phase lag index (W); 10 × 9/2 channel pairs × 12 frequency bands]. Henceforth, we used the following naming convention for the features: <feature-type>-<channel or channel pair>-<frequency band>. For example, we denoted the log power in the C3 channel in the low-delta band as LP-C3-Lδ and the phase synchrony between the C3 and F3 channels in the high-beta band as W-C3-F3-Hβ.

### Feature Processing

**Figure 2:**
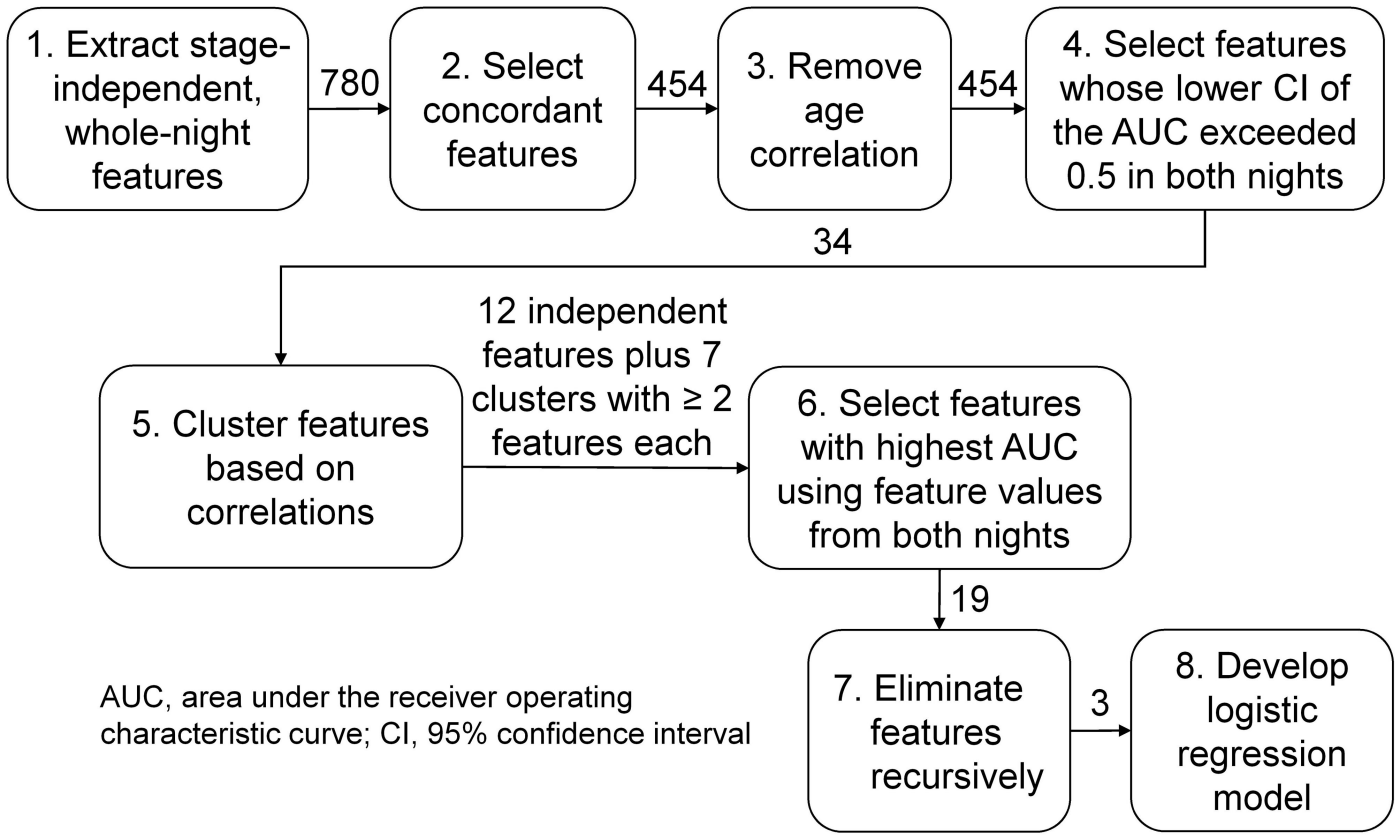
Analysis workflow for feature selection and classifier development. We analyzed three types of sleep-stage independent features, (1) log powers, (2) their coefficients of variation, and (3) the phase synchrony between pairs of electroencephalography channels, averaged across the entire night, in twelve frequency bands of interest. We started with 780 features (120 type 1, 120 type 2, and 540 type 3) and ended up with three features, one of type 1 and two of type 3.

We performed the following operations to process the features for use in a multivariate classifier. First, to avoid problems due to heteroscedasticity during classifier development, we log-transformed the synchrony features to scale them similarly to the log features LP and LCV. Second, we used the concordance correlation coefficient (19) to assess the consistency of feature values across the two consecutive nights in the *training* set, and retained features with a correlation that exceeded 0.7 (Figure 2, step 2). Third, to preclude confounding effects due to age (20), we first computed Pearson's correlation between each feature and age for subjects without PTSD in the *training* set. [It should be noted that we used only subjects without PTSD because determining correlation coefficients based on subjects with PTSD could potentially remove disorder-related changes (21)]. Then, for each feature that was significantly correlated with age (*p* < 0.05), we estimated the parameters of a linear regression model relating age to that feature and used them to statistically remove the effect of age in the entire population (22) (i.e., all subjects in the *training* and *test* sets; Figure 2, step 3).

### Univariate Feature Selection and Clustering

Following the processing steps, we performed a univariate analysis to select features discriminative of PTSD in the *training* set using the area under the receiver operating characteristic (ROC) curve (AUC) as the metric. For each feature, we computed the AUC and the corresponding lower and upper bounds of the 95% confidence interval (CI) and selected features for which the lower bound of the CI exceeded 0.5 on each of the two nights to guard against chance associations (Figure 2, step 4). For all subsequent steps of the analysis workflow (Figure 2, steps 5–8), we concatenated the feature values from each of the two nights of the *training* data into a single vector for each feature. To identify and remove correlated features, we clustered the feature vectors using a dendrogram with the distance correlation (23) as a metric. Whereas, a Pearson's correlation of 0 between two features only indicates uncorrelatedness in a linear sense, a distance correlation of 0 between two features indicates that they are independent. Also, unlike Pearson's correlation, which can take values between −1 and +1, the distance correlation ranges between 0 (independent) and 1 (perfect linear correlation). We performed the clustering step because training a classifier using correlated features can result in overfitting, due to the increased number of redundant parameters in the model, and reduced performance on the *test* set. To avoid these problems, we grouped features with a distance correlation exceeding 0.7 into a cluster (Figure 2, step 5).

### Multivariate Feature Selection and Classifier Development

For each cluster consisting of two or more features, we chose the feature with the highest AUC of the concatenated feature vector, as the representative feature for that cluster. To these (cluster-derived) features, we added the remaining (independent) features, which did not group into any of the clusters, for classifier development (Figure 2, step 6). Subsequently, to further reduce the chance of overfitting, we performed recursive feature elimination (24) via six-fold cross validation with a logistic regression model to obtain the smallest set of features for which the regression coefficients were non-zero (Figure 2, step 7).

### Model Development and Evaluation

We developed the logistic regression model using the smallest feature set on the combined *training* data from both nights, and assessed model performance on data from each of the two nights of the *test* set. We evaluated model performance by using the AUC score and by computing sensitivity, specificity, and positive predictive value (PPV) of the model predictions for different threshold values. Unlike sensitivity and specificity, the PPV depends on the prevalence of PTSD in a given population. In the present study population, the prevalence of PTSD [39% (31 of 78 subjects)] was higher than the estimated value (~15%) in the overall population of combat-exposed Veteran men (25–27). Therefore, to avoid overestimating the PPV, we re-wrote the standard formula so that the PPV (28), henceforth termed as “adjusted PPV” is an explicit function of the prevalence of PTSD in the overall population (see Note A in the Supplementary materials).

We performed all of the aforementioned analyses via custom scripts written in MATLAB (The MathWorks Inc., Natick, MA), as well as in Python version 3.5.2 using the numpy, scipy, sklearn, and pandas libraries.

## Results

### Feature Selection

**Figure 3:**
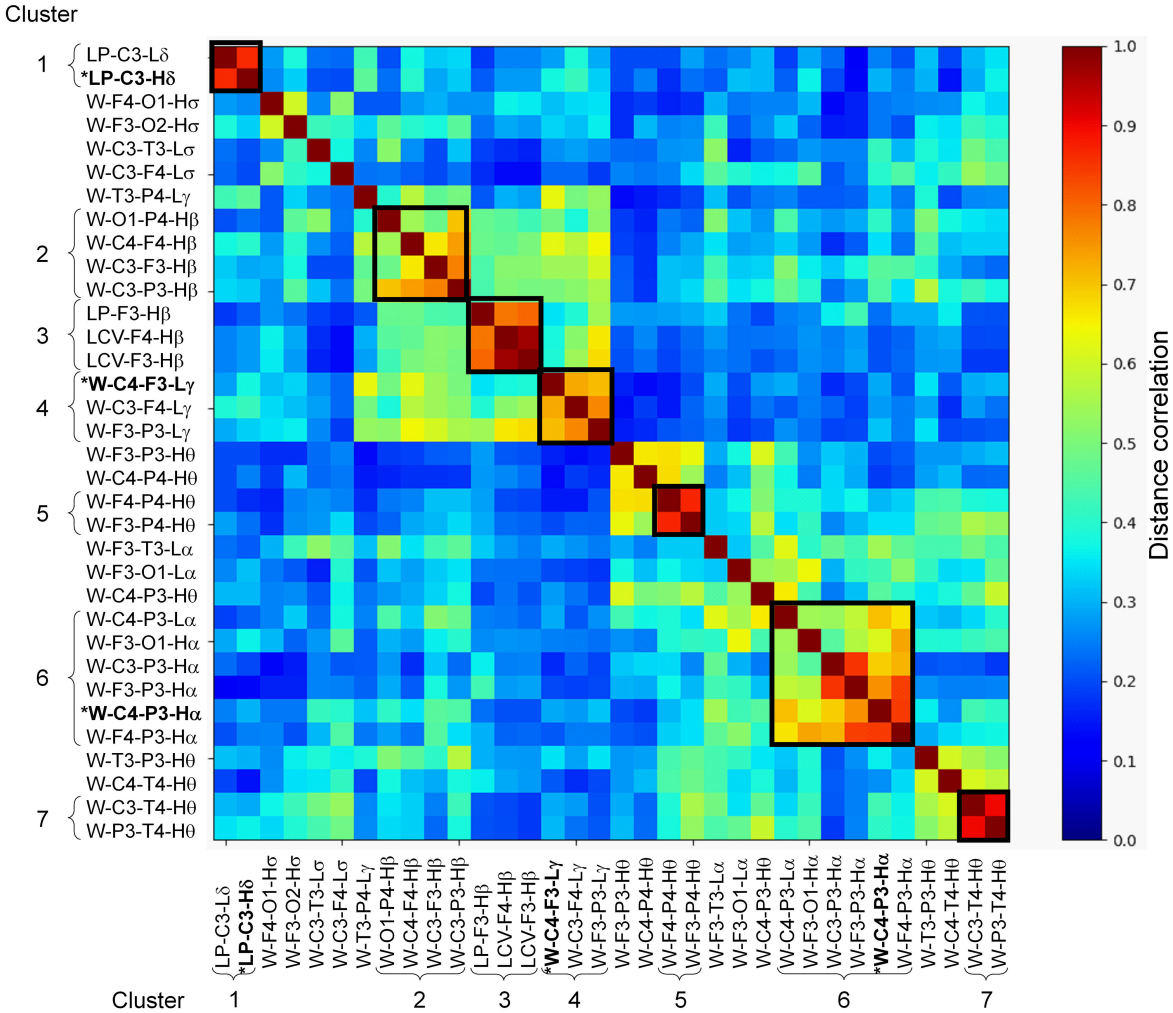
Distance correlation between the 34 stage-independent, whole-night features extracted from 10 electroencephalography channels across both nights of the *training* set (those obtained after step 4 in Figure 2). Dendrogram clustering revealed seven clusters with correlation values exceeding 0.7 (dark squares in the image, where the curly brackets identify the features within a cluster). Features marked with asterisk and highlighted in bold-face text (one each in clusters 1, 4, and 6) indicate the three features selected via recursive feature elimination. The 12 independent features obtained after step 5 in Figure 2 are located between the seven clusters.

Of the 780 features computed for each subject, 454 were concordant (i.e., the concordance correlation coefficient exceeded 0.7) across the two consecutive nights of recordings on the *training* set (Figure 2). Among these, 86 features were significantly correlated with age in subjects without PTSD in the *training* set (*n* = 29), which were then corrected to remove age effects from our study population. We then computed the univariate AUCs and the corresponding 95% CIs for each of the 454 features, and selected the 34 for which the lower bound of the CI exceeded 0.5 in each of the two nights of the *training* set. As noted above in the Methods Section, for all subsequent processing steps (Figure 2, steps 5–8), we concatenated the feature values from the two nights of the *training* data to form a single vector for each feature. Distance correlation-based clustering revealed seven clusters with two or more features each (with distance correlation > 0.7) plus 12 independent features that did not cluster with any other feature (Figure 3). For each of the seven clusters, we then selected the feature with the highest AUC as the representative of that cluster, forming a total of 19 (7 + 12) independent features for further analysis.

### Model Development and Evaluation

Using the concatenated vectors for the 19 features, we performed recursive feature elimination using the logistic regression model, which resulted in a final model consisting of three features with non-zero model coefficients (Figure 2, step 8). One of the features was LP-C3-Hδ, whereas the other two were phase synchronies in the high-alpha and low-gamma bands. We provide their values and group differences in Supplementary Tables S2–S4. The final model, combining these three features, yielded a *training*-set AUC of 0.83 on the combined data from the two nights and *test*-set AUCs of 0.84 for Night 1 and 0.80 for Night 2 (Table 3). These values were considerably larger than the univariate *test*-set AUCs of any of the three features, which ranged from 0.55 to 0.74 across the two nights (Table 4), indicating superior performance for the multivariate classifier.

**Table 3:** Area under the receiver operating characteristic curve (AUC) for a logistic regression model consisting of the three features shown in Table 4 and developed by combining data from both nights of the *training* set.

	**Training**	**Testing**	
		**Night 1**	**Night 2**
AUC	0.83 (0.73, 0.92)	0.84 (0.70, 0.98)	0.80 (0.64, 0.96)
Threshold = 0.37 (Training Sen. = 0.81)			
Sen.	0.81	0.62	0.54
Spe.	0.74	0.89	0.67
Adj. PPV	0.35	0.50	0.22
Threshold = 0.26 (Training Sen. = 0.92)			
Sen.	0.92	0.85	0.85
Spe.	0.57	0.67	0.67
Adj. PPV	0.27	0.31	0.31

*Values within parentheses indicate 95% confidence intervals*.

*Adj. PPV = [sensitivity × prevalence] / [(sensitivity × prevalence) + {(1 - specificity) × (1 - prevalence)}]*.

*The sensitivity (Sen.), specificity (Spe.), and adjusted positive predictive value (Adj. PPV) for a PTSD prevalence of 15% at two different thresholds of the model output (corresponding to training-set sensitivities of 0.81 and 0.92) are shown above*.

**Table 4:** Area under the receiver operating characteristic curve (AUC) for each of the three features used in the logistic regression model.

**Feature**	**Training set [*n* = 47 (18 PTSD)]**	**Test set [*****n*** **=** **31 (13 PTSD)]**	
		**Night 1**	**Night 2**
LP-C3-Hδ			
AUC	**0.71** (0.60, 0.82)	0.63 (0.42, 0.84)	0.60 (0.38, 0.82)
Threshold = +1.28 (Training Sen. = 0.92)			
Sen.	0.92	0.77	0.77
Spe.	0.26	0.28	0.33
Adj. PPV	0.18	0.16	0.17
W-C4-P3-Hα			
AUC	**0.74** (0.63, 0.84)	0.55 (0.34, 0.77)	0.64 (0.42, 0.86)
Threshold = −1.62 (Training Sen. = 0.92)			
Sen.	0.92	1.00	0.92
Spe.	0.26	0.50	0.33
Adj. PPV	0.18	0.26	0.20
W-C4-F3-Lγ			
AUC	**0.73** (0.61, 0.84)	0.67 (0.47, 0.88)	**0.74** (0.57, 0.92)
Threshold = −2.23 (Training Sen. = 0.92)			
Sen.	0.92	0.85	1.00
Spe.	0.19	0.11	0.06
Adj. PPV	0.17	0.14	0.16

*Values within parentheses indicate 95% confidence intervals (CIs). Bold values indicate statistical significance (lower bound of the CI > 0.50)*.

*Adj. PPV = [sensitivity × prevalence] / [(sensitivity × prevalence) + {(1–specificity) × (1–prevalence)}]*.

*For each feature, the sensitivity (Sen.), specificity (Spe.), and adjusted positive predictive value (Adj. PPV) for a PTSD prevalence of 15% at a training-set sensitivity of 0.92 are shown above the AUC values*.

We used the ROC curve of the regression model outcome on the *training* set to search for two thresholds that correspond to sensitivity values above 0.80 and 0.90. This search yielded thresholds of 0.37 and 0.26, which corresponded to *training*-set sensitivities of 0.81 and 0.92, respectively. At the threshold of 0.37, the model yielded *test*-set sensitivities that were much lower than the *training*-set sensitivity (0.62 on Night 1 and 0.54 on Night 2 for the *test* set vs. 0.81 for the *training* set; Table 3). In contrast, at the 0.26 threshold, the *test*-set sensitivity was 0.85 on each of the two nights (Table 3), while the specificities were 18% higher than those of the *training* set (0.67 on each of the two nights for the *test* set vs. 0.57 for the *training* set; Table 3). Importantly, the adjusted PPV was 0.31 for the *test* set, which was twice the PTSD prevalence value of 0.15 in combat-exposed Veteran men (Table 3).

At thresholds corresponding to a *training*-set sensitivity of 0.92 for each of the three individual features used in the model, the univariate *test*-set sensitivities ranged from 0.77 to 1.00 over the two consecutive nights (Table 4), which were comparable to the sensitivity of the regression model (0.85; Table 3). However, the *test*-set specificities (range: 0.06–0.33; Table 4) and adjusted PPVs (range: 0.14–0.26; Table 4) were much smaller than the corresponding values for the regression model (specificity = 0.67, adjusted PPV = 0.31; Table 3). These results further underscore the advantage of combining the three features to identify individuals with PTSD.

## Discussion

We found that a logistic regression model, using three stage-independent, whole-night features, could discriminate subjects with and without PTSD at an individual level. First, we divided the study data into a *training* set, consisting of the first 47 consecutive subjects in the study (18 with PTSD), and a *testing* set, consisting of the next 31 subjects (13 with PTSD). Then, using only the *training* set, we identified three uncorrelated EEG features that discriminated subjects with and without PTSD in each of the two nights of the study (high-delta power in the C3 channel, phase synchrony between the C4 and P3 channels in the high-alpha band, and phase synchrony between the C4 and F3 channels in the low-gamma band). Using these features, we developed a logistic regression model based on the subjects from the *training* set. Then, to independently assess the performance of this model, we computed the value of the three features for each of the subjects in the *testing* set, and used the model to classify the 31 subjects in this set into PTSD or non-PTSD. Assessment of the logistic regression model on the *testing*-set data resulted in AUCs above 0.80 for each of the two consecutive nights, a high sensitivity (0.85), a moderate specificity (0.67), and an adjusted PPV of 0.31, which is twice the prevalence of PTSD in combat-exposed Veteran men (Table 3). This means that if the model classifies a combat-exposed Veteran man as having PTSD, the probability that the subject actually has this disorder is twice as large as a random-choice selection.

### Interpretation of the Three Selected Features

The three features used in the logistic regression model were from the delta-, alpha-, and gamma-band clusters (Figure 3; clusters 1, 6, and 4, respectively). Of these, the log powers in the C3 channel in both low- and high-delta bands (Figure 3; clusters 1) were smaller in subjects with PTSD compared to those without PTSD (Supplementary Table 1 shows the effect sizes for each feature, with negative effect sizes indicating lower feature values in PTSD.) These results are similar to those of our prior study, which showed that delta power during NREM is smaller in subjects with PTSD compared to those without PTSD (6). This is not surprising because, as noted in Methods section Feature Computation, sleep predominantly consists of the NREM stage 2 (Table 2) and, hence, any feature based on whole-night, stage-independent averages will contain the most information for this sleep stage. Given that delta power indicates sleep depth (29), it is likely that lower delta power in subjects with PTSD indicates disturbed sleep.

The first of the two synchrony features, W-C4-P3-Hα, was the representative of the alpha-band cluster (Figure 3, cluster 6), which mainly consisted of synchronies between EEG channels located on the left hemisphere, save for two synchrony pairs that involved the C4 channel. The synchronies in this cluster were larger in subjects with PTSD compared to those without PTSD (Supplementary Table 1). This is also in line with our prior findings, in which subjects with PTSD showed larger alpha synchrony than subjects without PTSD in the left fronto-parietal regions during NREM sleep (8). The second feature, W-C4-F3-Lγ, was the representative of the gamma-band cluster (Figure 3, cluster 4), which mainly consisted of cross-hemisphere synchronies between the frontal and central channels that were larger in subjects with PTSD (Supplementary Table 1). Although the increased synchrony in these bands may reflect impaired sleep processes in PTSD, focused research on this topic will be needed before we can make any conclusive statements regarding the specific underlying neurophysiological mechanisms of these features.

### Model Evaluation Procedure

In general, there are two main approaches to evaluate the performance of classification models. One approach is cross-validation, which entails multiple rounds of model development and evaluation on different partitions of the study data. The other involves splitting the data into a *training* set and a *test* set at the outset. The former approach allows for the use of the entire dataset for model development, but because each subject is used in model development in at least one of the cross-validation rounds, its ability to truly assess model performance on unseen subjects may be reduced. Although the latter approach decreases the sample size available for model development, it allows for independent evaluation of model performance. In this work, we used this approach because it more closely mimics how the results in one study are subsequently validated in future studies using a completely different set of subjects.

### Limitations of the Study

A limitation of this work is that our study population excluded subjects with PTSD who had comorbid sleep disorders, such as depression (30) or insomnia (31), which share symptoms with PTSD. To test whether the features included in the logistic regression model are specific to PTSD, we would need to test the model in two different populations: one that included sleep disorders other than PTSD and another that included subjects with PTSD and comorbidities. If the features were specific to PTSD, then the model performance would be degraded in the first population and improved in the second. However, the model performance in the second population should not be as good as those of the present study population, which consisted of comorbidity-free subjects with PTSD.

Another potential limitation relates to the use of whole-night averages of the EEG features rather than an approach that considers the time-series nature of the EEG signal. By averaging the features across the entire 8 h of time in bed, it is possible that our analysis excluded alterations in the power or synchrony features in short-length sleep stages, e.g., during REM or NREM stage 3 sleep. However, analyzing time-series features in a naïve manner, i.e., by assuming that the feature value at one time point is independent of that at another time point, could increase the chance of false associations when sample sizes are small, as was the case in this study. A robust analysis of time-series features would require identification of temporal patterns in each feature (32) and, hence, a more elaborate methodology whose results would likely be difficult to compare with other studies.

## Conclusion

In this work, we assessed the ability of a multivariate classifier to diagnose PTSD at an individual level, using whole-night, stage-independent features to obviate the need for laborious manual scoring of sleep. After identifying univariate features associated with PTSD, we combined them into a logistic regression model to test whether the model could discriminate subjects with and without PTSD. We developed the model on an initial *training* set from consecutive subjects enrolled in the study, and then evaluated its performance on a separate, independent *test* set from subsequent subjects. Performance on the *test* set yielded AUCs above 0.80 for each of the two consecutive nights, high sensitivity (0.85), and an adjusted PPV that is twice the prevalence of PTSD in combat-exposed Veteran men. These findings imply that, if the model predicts that a subject has PTSD, the likelihood of that subject actually having PTSD is twice the underlying prevalence rate. Thus, the model provides an objective means to more accurately identify individuals with this disorder.

## Data Availability Statement

The datasets presented for this study are available on request to the corresponding author.

## Ethics Statement

The studies involving human participants were reviewed and approved by University of Pittsburgh Institutional Review Board (Pittsburgh, PA) The U.S. Army Medical Research and Development Command Human Research Protection Office (Ft. Detrick, MD). The patients/participants provided their written informed consent to participate in this study.

## Author Contributions

SL, CW, and JR conceived the research idea and study objectives. SL performed all analyses reported in the study. SL and TO wrote the manuscript. CW and JR provided inputs for improving the analysis and edited the manuscript. JC and AG performed the laboratory study. All authors read and approved the manuscript.

## Conflict of Interest

The authors declare that the research was conducted in the absence of any commercial or financial relationships that could be construed as a potential conflict of interest.

## References

[1] Germain A. Sleep disturbances as the hallmark of PTSD: where are we now? Am J Psychiatry. (2013) 170:372–82. 10.1176/appi.ajp.2012.12040432 23223954 PMC4197954

[2] Neylan TC Lenoci M Maglione ML Rosenlicht NZ Metzler TJ Otte C. Delta sleep response to metyrapone in post-traumatic stress disorder. Neuropsychopharmacology. (2003) 28:1666–76. 10.1038/sj.npp.1300215 12799616

[3] Mellman TA Pigeon WR Nowell PD Nolan B. Relationships between REM sleep findings and PTSD symptoms during the early aftermath of trauma. J Trauma Stress. (2007) 20:893–901. 10.1002/jts.20246 17955526

[4] Cohen DJ Begley A Alman JJ Cashmere DJ Pietrone RN Seres RJ. Quantitative electroencephalography during rapid eye movement (REM) and non-REM sleep in combat-exposed veterans with and without post-traumatic stress disorder. J Sleep Res. (2013) 22:76–82. 10.1111/j.1365-2869.2012.01040.x 22845675 PMC3488164

[5] Cowdin N Kobayashi I Mellman TA. Theta frequency activity during rapid eye movement (REM) sleep is greater in people with resilience vs. PTSD. Exp Brain Res. (2014) 232:1479–85. 10.1007/s00221-014-3857-5 24531640 PMC4449337

[6] Wang C Ramakrishnan S Laxminarayan S Dovzhenok A Cashmere JD Germain A. An attempt to identify reproducible high-density EEG markers of PTSD during sleep. Sleep. (2020) 43:zsz207. 10.1093/sleep/zsz207 31553047

[7] Vinck M Oostenveld R van Wingerden M Battaglia F Pennartz CM. An improved index of phase-synchronization for electrophysiological data in the presence of volume-conduction, noise and sample-size bias. Neuroimage. (2011) 55:1548–65. 10.1016/j.neuroimage.2011.01.055 21276857

[8] Laxminarayan S Wang C Ramakrishnan S Oyama T Cashmere DJ Germain A. Alterations in sleep EEG synchrony in combat-exposed veterans with PTSD. Sleep. (2020) 43:zsaa006. 10.1093/sleep/zsaa006 31971594 PMC8240478

[9] Modarres MH Opel RA Weymann KB Lim MM. Strong correlation of novel sleep electroencephalography coherence markers with diagnosis and severity of posttraumatic stress disorder. Sci Rep. (2019) 9:4247. 10.1038/s41598-018-38102-4 30862872 PMC6414519

[10] Blake DD Weathers FW Nagy LM Kaloupek DG Gusman FD Charney DS. The development of a clinician-administered PTSD scale. J Trauma Stress. (1995) 8:75–90. 10.1002/jts.2490080106 7712061

[11] Bastien CH Vallieres A Morin CM. Validation of the insomnia severity index as an outcome measure for insomnia research. Sleep Med. (2001) 2:297–307. 10.1016/S1389-9457(00)00065-4 11438246

[12] Lehrner A Yehuda R. Biomarkers of PTSD: military applications and considerations. Eur J Psychotraumatol. (2014) 5:27397. 10.3402/ejpt.v5.23797 PMC4138702 25206945

[13] Buysse DJ Reynolds CF 3rd Monk TH Berman SR Kupfer DJ. The pittsburgh sleep quality index: a new instrument for psychiatric practice and research. Psychiatry Res. (1989) 28:193–213. 10.1016/0165-1781(89)90047-4 2748771

[14] Lowe B Kroenke K Herzog W Grafe K. Measuring depression outcome with a brief self-report instrument: sensitivity to change of the patient health questionnaire (PHQ-9). J Affect Disord. (2004) 81:61–6. 10.1016/S0165-0327(03)00198-8 15183601

[15] Richards A Metzler TJ Ruoff LM Inslicht SS Rao M Talbot LS. Sex differences in objective measures of sleep in post-traumatic stress disorder and healthy control subjects. J Sleep Res. (2013) 22:679–87. 10.1111/jsr.12064 23763708 PMC3958933

[16] Silber MH Ancoli-Israel S Bonnet MH Chokroverty S Grigg-Damberger MM Hirshkowitz M. The visual scoring of sleep in adults. J Clin Sleep Med. (2007) 3:121–31. 10.5664/jcsm.26814 17557422

[17] Brunner DP Vasko RC Detka CS Monahan JP Reynolds CF 3rd Kupfer DJ. Muscle artifacts in the sleep EEG: automated detection and effect on all-night EEG power spectra. J Sleep Res. (1996) 5:155–64. 10.1046/j.1365-2869.1996.00009.x 8956205

[18] Liu J Ramakrishnan S Laxminarayan S Neal M Cashmere DJ Germain A. Effects of signal artefacts on electroencephalography spectral power during sleep: quantifying the effectiveness of automated artefact-rejection algorithms. J Sleep Res. (2018) 27:98–102. 10.1111/jsr.12576 28656650

[19] Lin LI. A concordance correlation coefficient to evaluate reproducibility. Biometrics. (1989) 45:255–68. 10.2307/2532051 2720055

[20] Carrier J Land S Buysse DJ Kupfer DJ Monk TH. The effects of age and gender on sleep EEG power spectral density in the middle years of life (ages 20–60 years old). Psychophysiology. (2001) 38:232–42. 10.1111/1469-8986.3820232 11347869

[21] Dukart J Schroeter ML Mueller K Alzheimer's Disease Neuroimaging Initiative. Age correction in dementia–matching to a healthy brain. PLoS ONE. (2011) 6:e22193. 10.1371/journal.pone.0022193 21829449 PMC3146486

[22] Green PJ. Iteratively reweighted least squares for maximum likelihood estimation, and some robust and resistant alternatives. J R Stat Soc Series B. (1984) 46:149–170. 10.1111/j.2517-6161.1984.tb01288.x

[23] Szekely GJ Rizzo ML Bakirov NK. Measuring and testing dependence by correlation of distances. Ann Statist. (2007) 35:2769–94. 10.1214/009053607000000505

[24] Zhu J Hastie T. Classification of gene microarrays by penalized logistic regression. Biostatistics. (2004) 5:427–43. 10.1093/biostatistics/kxg046 15208204

[25] Kulka RA Schlenger WE Fairbank JA Hough RL Jordan BK Marmar CR. Trauma and the Vietnam War Generation: Report of Findings from the National Vietnam Veterans Readjustment Study. Philadelphia, PA: Brunner/Mazel (1990).

[26] Kang HK Natelson BH Mahan CM Lee KY Murphy FM. Post-traumatic stress disorder and chronic fatigue syndrome-like illness among Gulf War veterans: a population-based survey of 30,000 veterans. Am J Epidemiol. (2003) 157:141–8. 10.1093/aje/kwf187 12522021

[27] Tanielian TL Jaycox L. Rand Corporation. Invisible Wounds of War : Psychological and Cognitive Injuries, their Consequences, and Services to Assist Recovery. Santa Monica, CA: RAND (2008). 10.1037/e527612010-001

[28] Linn S Grunau PD. New patient-oriented summary measure of net total gain in certainty for dichotomous diagnostic tests. Epidemiol Perspect Innov. (2006) 3:11. 10.1186/1742-5573-3-11 17022816 PMC1635036

[29] Neckelmann D Ursin R. Sleep stages and EEG power spectrum in relation to acoustical stimulus arousal threshold in the rat. Sleep. (1993) 16:467–77. 8378687

[30] Leistedt SJJ Coumans N Dumont M Lanquart J-P Stam CJ Linkowski P. Altered sleep brain functional connectivity in acutely depressed patients. Hum Brain Mapp. (2009) 30:2207–19. 10.1002/hbm.20662 18937282 PMC6870637

[31] Corsi-Cabrera M Figueredo-Rodríguez P del Río-Portilla Y Sánchez-Romero J Galán L Bosch-Bayard J. Enhanced frontoparietal synchronized activation during the wake-sleep transition in patients with primary insomnia. Sleep. (2012) 35:501–11. 10.5665/sleep.1734 22467988 PMC3296792

[32] Geurts P. Pattern extraction for time series classification. In: deRaedt L Seibes A, editors. Principles of Data Mining and Knowledge Discovery. PKDD 2001 Lecture Notes in Computer Science 2168. Berlin: Springer-Verlag. (2001). 10.1007/3-540-44794-6_10

