# Supplementary material for: Identification of Veterans With PTSD Based on EEG Features Collected During Sleep
Source: Front Psychiatry. 2020 Oct 30;11:532623. doi: 10.3389/fpsyt.2020.532623 (PMC7673410; doi:10.3389/fpsyt.2020.532623)
Supplement: Supplementary file 1 [file Data_Sheet_1.pdf]

Supplementary materials

Note A: Formula for the positive predictive value (PPV) adjusted for the prevalence in a population

A binary classifier is evaluated using the confusion matrix shown below.

	Ground truth is positive	Ground truth is negative
Model predicts positive	True positives (TP)	False positives (FP)
Model predicts negative	False negatives (FN)	True negatives (TN)
	All positives (P) = TP + FN	All negatives (N) = FP + TN

Here, sensitivity is given by TP/P and specificity by TN/N. Note that, because each of these measures only depends on one of the two classes, they are not affected by the prevalence rate in the study population. In contrast, the PPV is affected by the prevalence rate of the positive condition in the study population, because the PPV depends on both classes [PPV = (TP) / (TP + FP)]. However, we can re-write this equation so that the PPV becomes explicitly dependent on the prevalence and use the overall prevalence in the population instead of the study prevalence.

We can then write, $\mathrm{PPV} = \dfrac{\frac{\mathrm{TP}}{\mathrm{P+N}}}{\frac{\mathrm{TP}}{\mathrm{P+N}} + \frac{\mathrm{FP}}{\mathrm{P+N}}}$

$$= \frac{\frac{\mathrm{TP}}{\mathrm{P}} \times \frac{\mathrm{P}}{\mathrm{P+N}}}{\frac{\mathrm{TP}}{\mathrm{P}} \times \frac{\mathrm{P}}{\mathrm{P+N}} + \frac{\mathrm{FP}}{\mathrm{N}} \times \frac{\mathrm{N}}{\mathrm{P+N}}}$$

Noting that TP/P is the sensitivity, P/(P+N) is the prevalence, FP/N is (1 – specificity), and N/(P+N) is (1 – prevalence), we derive the following formula:

$$\text{Adjusted PPV} = \frac{(\text{sensitivity} \times \text{prevalence})}{(\text{sensitivity} \times \text{prevalence}) + \{(1 - \text{specificity}) \times (1 - \text{prevalence})\}}$$

,where the PPV is an explicit function of the prevalence of PTSD in the overall population of combat-exposed Veteran men, i.e., 15% (26-28).

Table S1: Effect sizes, areas under the receiver operating characteristic curve (AUCs), and Wilcoxon rank-sum test *p*-values for the 34 features in the entire study population [n = 78 (31 with PTSD)], selected based on the criterion that the lower confidence interval of the AUC exceeded 0.5 on both nights for data from the *training* set alone [n = 47 (18 with PTSD)]. Cells shaded in grey indicate feature clusters. Unshaded cells indicate the 12 independent features. The three features used in the logistic regression model are highlighted in bold-faced text.

Cluster and Features	Night 1			Night 2		
	Effect size*	AUC	*p*-value	Effect size*	AUC	*p*-value
Cluster 1						
LP-C3-Lδ	-0.58 (-1.09, -0.04)	0.65 (0.52, 0.77)	0.02	-0.46 (-0.94, 0.02)	0.64 (0.50, 0.76)	0.04
**LP-C3-Hδ**	**-0.58 (-1.12, -0.10)**	**0.67 (0.53, 0.78)**	**0.01**	**-0.65 (-1.09, -0.14)**	**0.67 (0.53, 0.79)**	**0.01**
W-F4-O1-Hσ	0.73 (0.23, 1.33)	0.69 (0.55, 0.80)	0.01	0.76 (0.26, 1.30)	0.70 (0.56, 0.80)	<0.01
W-F3-O2-Hσ	0.48 (-0.02, 0.99)	0.62 (0.48, 0.74)	0.07	0.46 (-0.02, 0.96)	0.62 (0.48, 0.74)	0.07
W-C3-T3-Lσ	0.42 (-0.05, 0.90)	0.62 (0.49, 0.74)	0.07	0.35 (-0.19, 0.78)	0.59 (0.45, 0.71)	0.20
W-C3-F4-Lσ	0.61 (0.18, 1.14)	0.69 (0.55, 0.80)	0.01	0.60 (0.10, 1.18)	0.66 (0.53, 0.77)	0.02
W-T3-P4-Lγ	0.68 (0.14, 1.18)	0.68 (0.55, 0.79)	0.01	0.52 (0.02, 1.01)	0.64 (0.50, 0.75)	0.04
Cluster 2						
W-O1-P4-Hβ	0.59 (0.11, 1.13)	0.65 (0.51, 0.76)	0.03	0.97 (0.39, 1.55)	0.75 (0.63, 0.85)	<0.01
W-C4-F4-Hβ	0.60 (0.13, 1.27)	0.67 (0.53, 0.78)	0.01	0.64 (0.14, 1.27)	0.66 (0.53, 0.77)	0.01
W-C3-F3-Hβ	0.62 (0.15, 1.29)	0.67 (0.53, 0.78)	0.01	0.55 (0.06, 1.20)	0.65 (0.52, 0.76)	0.03
W-C3-P3-Hβ	0.57 (0.10,1.29)	0.65 (0.51, 0.77)	0.03	0.41 (-0.08, 0.94)	0.61 (0.47, 0.73)	0.11
Cluster 3						
LP-F3-Hβ	0.51 (0.09, 0.95)	0.64 (0.51,0.76)	0.04	0.60 (0.16, 1.02)	0.66 (0.53, 0.77)	0.02
LCV-F4-Hβ	-0.63 (-1.17, -0.10)	0.66 (0.53, 0.77)	0.02	-0.62 (-1.31, -0.10)	0.65 (0.52, 0.76)	0.02
LCV-F3-Hβ	-0.63 (-1.10, -0.10)	0.65 (0.52, 0.76)	0.03	-0.63 (-1.10, -0.10)	0.63 (0.50, 0.75)	0.05
Cluster 4						
**W-C4-F3-Lγ**	**0.84 (0.33, 1.40)**	**0.70 (0.57, 0.81)**	**<0.01**	**0.90 (0.42, 1.46)**	**0.74 (0.60, 0.84)**	**<0.01**

W-C3-F4-Lγ	0.53<br>(-0.02, 0.97)	0.62<br>(0.49, 0.74)	0.07	0.87<br>(0.33, 1.43)	0.71<br>(0.58, 0.82)	<0.01
W-F3-P3-Lγ	0.63<br>(0.09, 1.25)	0.65<br>(0.52, 0.77)	0.02	0.94<br>(0.36, 1.76)	0.72<br>(0.58, 0.82)	<0.01
W-F3-P3-Hθ	0.58<br>(0.07, 1.10)	0.65<br>(0.52, 0.77)	0.02	0.33<br>(-0.14, 0.85)	0.59<br>(0.46, 0.71)	0.17
W-C4-P4-Hθ	0.38<br>(-0.06, 0.90)	0.62<br>(0.48, 0.73)	0.08	0.33<br>(-0.09, 0.79)	0.61<br>(0.48, 0.73)	0.09
Cluster 5						
W-F4-P4-Hθ	0.58<br>(0.09, 1.32)	0.65<br>(0.50, 0.76)	0.03	0.73<br>(0.12, 1.25)	0.67<br>(0.54, 0.79)	0.01
W-F3-P4-Hθ	0.83<br>(0.20, 1.51)	0.70<br>(0.56, 0.81)	<0.01	0.60<br>(0.06, 1.08)	0.67<br>(0.53, 0.78)	0.01
W-F3-T3-Lα	0.66<br>(0.21, 1.13)	0.68<br>(0.54, 0.78)	0.01	0.60<br>(0.06, 1.06)	0.65<br>(0.52, 0.77)	0.02
W-F3-O1-Lα	0.67<br>(0.21, 1.30)	0.70<br>(0.56, 0.80)	<0.01	0.56<br>(0.14, 1.14)	0.67<br>(0.54, 0.78)	0.01
W-C4-P3-Hθ	0.59<br>(0.13, 1.19)	0.67<br>(0.53, 0.78)	0.01	0.68<br>(0.22, 1.27)	0.68<br>(0.55, 0.79)	0.01
Cluster 6						
W-C4-P3-Lα	0.84<br>(0.36, 1.54)	0.72<br>(0.59, 0.83)	<0.01	0.59<br>(0.17, 1.14)	0.67<br>(0.54, 0.78)	0.01
W-F3-O1-Hα	0.86<br>(0.38, 1.44)	0.74<br>(0.61, 0.84)	<0.01	0.95<br>(0.44, 1.57)	0.74<br>(0.61, 0.84)	<0.01
W-C3-P3-Hα	0.63<br>(0.11, 1.21)	0.68<br>(0.54, 0.79)	0.01	0.55<br>(0.03, 1.07)	0.64<br>(0.51, 0.76)	0.03
W-F3-P3-Hα	0.72<br>(0.21, 1.29)	0.69<br>(0.55, 0.80)	<0.01	0.72<br>(0.26, 1.24)	0.70<br>(0.57, 0.80)	<0.01
**W-C4-P3-Hα**	**0.99**<br>**(0.48, 1.67)**	**0.76**<br>**(0.63, 0.85)**	**<0.01**	**0.91**<br>**(0.46, 1.41)**	**0.76**<br>**(0.63, 0.85)**	**<0.01**
W-F4-P3-Hα	0.89<br>(0.40, 1.43)	0.73<br>(0.60, 0.83)	<0.01	0.82<br>(0.34, 1.43)	0.73<br>(0.60, 0.83)	<0.01
W-T3-P3-Hθ	0.63<br>(0.11, 1.19)	0.66<br>(0.52, 0.77)	0.02	0.51<br>(0.05, 1.09)	0.63<br>(0.50, 0.75)	0.05
W-C4-T4-Hθ	0.58<br>(0.03, 1.11)	0.64<br>(0.51, 0.76)	0.03	0.48<br>(0.07, 0.98)	0.64<br>(0.51, 0.76)	0.03
Cluster 7						
W-C3-T4-Hθ	0.57<br>(0.03, 1.03)	0.64<br>(0.50, 0.75)	0.04	0.53<br>(0.08, 1.07)	0.66<br>(0.53, 0.77)	0.02
W-P3-T4-Hθ	0.63<br>(0.22, 1.10)	0.67<br>(0.54, 0.78)	0.01	0.72<br>(0.32, 1.21)	0.71<br>(0.58, 0.81)	<0.01

Values within parentheses indicate 95% confidence intervals.
*Robust Cohen's *d* computed using 20% trimmed means and 20% winsorized variances as the difference between values from subjects with and without PTSD.

Table S2: Training-set values of the three features used to develop the logistic regression model. Feature values for the 29 subjects without PTSD are listed first, followed by those for the 18 subjects with PTSD.

Subjects	LP-C3-Hδ		W-C4-P3-Hα		W-C4-F3-Lγ	
	Night 1	Night 2	Night 1	Night 2	Night 1	Night 2
Without PTSD						
1	1.14	1.04	-0.94	-1.00	-2.28	-2.02
2	1.19	1.26	-1.90	-1.76	-2.15	-2.29
3	1.17	1.19	-0.76	-0.92	-2.10	-2.15
4	1.60	1.66	-1.51	-1.46	-1.78	-1.74
5	1.47	1.55	-1.65	-1.59	-2.10	-2.14
6	1.24	1.32	-1.11	-0.97	-2.20	-1.98
7	1.18	1.03	-1.31	-1.33	-2.33	-2.23
8	1.38	1.32	-1.33	-1.37	-2.28	-2.23
9	1.06	1.12	-0.77	-0.92	-1.96	-2.15
10	1.03	0.93	-1.31	-1.51	-2.23	-2.32
11	1.06	0.99	-1.84	-1.54	-1.99	-1.78
12	0.97	1.12	-0.51	-0.57	-1.94	-2.12
13	1.09	1.11	-1.53	-1.62	-1.94	-2.11
14	1.27	1.31	-1.31	-1.02	-2.16	-2.29
15	1.07	1.13	-1.21	-1.16	-2.09	-1.94
16	1.13	1.17	-1.23	-1.42	-2.12	-2.34
17	1.25	1.41	-1.43	-1.78	-2.19	-2.08
18	1.21	1.20	-1.93	-1.69	-2.26	-2.21
19	0.94	1.02	-0.51	-0.51	-1.75	-1.86
20	1.50	1.55	-1.58	-0.87	-2.02	-2.10
21	1.20	1.26	-1.43	-1.97	-2.18	-2.13
22	1.38	1.34	-1.51	-1.90	-2.26	-2.21
23	1.18	1.11	-1.86	-1.99	-2.20	-2.01
24	1.19	1.07	-0.99	-1.11	-2.18	-2.14
25	1.32	1.21	-1.27	-1.20	-1.90	-1.93
26	1.11	1.14	-0.84	-1.07	-2.02	-2.14
27	0.94	1.05	-1.32	-1.68	-2.17	-2.29
28	1.23	1.30	-1.36	-1.19	-2.20	-2.19
29	1.11	1.13	-1.75	-1.76	-2.07	-2.05
With PTSD						
1	1.28	1.30	-0.76	-0.81	-2.15	-1.95
2	1.13	1.09	-1.15	-1.26	-1.95	-1.91
3	1.16	1.21	-1.63	-1.69	-1.91	-2.08
4	1.16	1.04	-0.84	-0.90	-1.90	-1.70

5	1.00	1.06	-1.27	-1.24	-1.88	-1.92
6	1.08	1.06	-0.45	-0.45	-2.15	-2.02
7	1.11	1.05	-0.56	-0.71	-2.07	-1.93
8	1.03	1.29	-0.56	-0.83	-1.87	-2.10
9	1.03	0.95	-1.58	-1.51	-2.10	-2.02
10	0.90	1.04	-0.83	-0.74	-2.23	-2.38
11	1.28	1.20	-0.70	-0.77	-1.31	-1.96
12	1.01	0.86	-0.80	-0.59	-1.90	-2.18
13	0.90	0.88	-1.10	-1.27	-1.72	-1.62
14	0.99	0.92	-0.55	-0.57	-1.63	-1.72
15	0.84	0.86	-1.05	-0.87	-2.09	-1.47
16	1.39	1.28	-1.45	-1.35	-2.41	-2.38
17	1.28	1.18	-0.97	-0.90	-1.98	-2.07
18	0.54	0.84	-1.81	-1.17	-1.83	-1.68

Table S3: Test-set values of the three features used to evaluate the performance of the logistic regression model. Feature values for the 18 subjects without PTSD are listed first, followed by those for the 13 subjects with PTSD.

Subjects	LP-C3-Hδ		W-C4-P3-Hα		W-C4-F3-Lγ	
	Night 1	Night 2	Night 1	Night 2	Night 1	Night 2
Without PTSD						
1	1.29	1.35	-0.99	-1.00	-2.10	-2.15
2	1.12	1.19	-1.63	-1.02	-1.95	-1.95
3	1.07	1.09	-1.87	-1.73	-2.16	-2.10
4	1.14	1.18	-1.75	-1.71	-2.05	-2.00
5	1.61	1.54	-1.43	-1.62	-2.07	-2.12
6	1.04	1.04	-1.53	-1.41	-2.22	-1.90
7	1.22	1.24	-1.18	-1.03	-2.03	-1.96
8	1.22	1.17	-1.64	-1.46	-1.67	-1.63
9	0.83	1.31	-1.86	-1.62	-2.19	-2.16
10	1.03	1.15	-0.96	-0.89	-2.13	-2.19
11	1.40	1.33	-1.40	-1.23	-2.15	-2.17
12	1.13	1.20	-1.11	-0.93	-1.82	-1.97
13	1.13	1.12	-2.00	-1.39	-2.07	-2.19
14	1.04	1.10	-1.66	-1.42	-2.12	-2.30
15	1.39	1.40	-1.44	-1.54	-2.02	-2.11
16	1.45	1.39	-1.80	-1.69	-2.24	-2.18
17	1.10	1.19	-1.64	-1.70	-2.11	-2.13
18	1.12	1.13	-1.60	-1.74	-2.31	-2.21
With PTSD						
1	1.10	1.22	-1.57	-1.37	-1.97	-2.08
2	1.07	1.22	-0.78	-0.75	-1.75	-1.76
3	1.17	1.23	-1.47	-1.30	-2.23	-2.13
4	1.12	1.18	-1.02	-0.72	-2.10	-2.09
5	1.13	1.08	-1.23	-1.82	-1.66	-1.86
6	1.01	1.16	-1.46	-1.27	-2.12	-2.00
7	1.02	0.96	-1.29	-1.44	-2.23	-2.16
8	1.01	0.96	-0.57	-0.57	-1.34	-1.07
9	1.46	1.40	-0.91	-0.62	-2.12	-2.04
10	1.31	1.33	-0.45	-0.47	-2.01	-2.07
11	0.85	0.83	-0.76	-1.08	-1.82	-1.75
12	0.90	1.01	-1.02	-1.19	-2.05	-2.03
13	1.32	1.45	-1.18	-0.98	-1.75	-1.87

Table S4: Group-average values of the three features used by the logistic regression model and the Wilcoxon rank-sum test *p*-values.

Features	Night 1			Night 2		
	Control	PTSD	*p*-value	Control	PTSD	*p*-value
Training set						
LP-C3-Hδ	1.19 (0.16)	1.06 (0.20)	**0.03**	1.21 (0.18)	1.06 (0.15)	**0.01**
W-C4-P3-Hα	-1.31 (0.39)	-1.00 (0.41)	**0.02**	-1.34 (0.40)	-0.98 (0.35)	**<0.01**
W-C4-F3-Lγ	-2.11 (0.15)	-1.95 (0.25)	**<0.01**	-2.11 (0.15)	-1.95 (0.24)	**<0.01**
Test set						
LP-C3-Hδ	1.19 (0.18)	1.11 (0.17)	0.24	1.23 (0.13)	1.16 (0.18)	0.37
W-C4-P3-Hα	-1.53 (0.30)	-1.05 (0.35)	**<0.01**	-1.40 (0.30)	-1.04 (0.40)	**0.02**
W-C4-F3-Lγ	-2.08 (0.15)	-1.93 (0.26)	0.11	-2.08 (0.16)	-1.92 (0.29)	**0.02**

Values within parentheses are standard deviations. Values in bold-faced text indicate $p < 0.05$.
